# A Comparative Evaluation of Sorption, Solubility, and Compressive Strength of Three Different Glass Ionomer Cements in Artificial Saliva: An *in vitro* Study

**DOI:** 10.5005/jp-journals-10005-1407

**Published:** 2017-02-27

**Authors:** Hind P Bhatia, Shivani Singh, Shveta Sood, Naresh Sharma

**Affiliations:** 1 Professor and Head, Department of Pedodontics and Preventive Dentistry, Manav Rachna Dental College, Faridabad, Haryana, India; 2 Student (3rd Year), Department of Pedodontics and Preventive Dentistry, Manav Rachna Dental College, Faridabad, Haryana, India; 3 Professor, Department of Pedodontics and Preventive Dentistry, Manav Rachna Dental College, Faridabad, Haryana, India; 4 Reader, Department of Pedodontics and Preventive Dentistry, Manav Rachna Dental College, Faridabad, Haryana, India

**Keywords:** Artificial saliva, Compressive strength, Glass Ionomer cements, Miracle mix, Solubility, Water sorption, Zirconomer.

## Abstract

**Aim:**

To evaluate and compare the sorption, solubility, and compressive strength of three different glass ionomer cements in artificial saliva - type IX glass ionomer cement, silver-reinforced glass ionomer cement, and zirconia-reinforced glass ionomer cement, so as to determine the material of choice for stress-bearing areas.

**Materials and methods:**

A total of 90 cylindrical specimens (4 mm diameter and 6 mm height) were prepared for each material following the manufacturer’s instructions. After subjecting the specimens to thermocycling, 45 specimens were immersed in artificial saliva for 24 hours for compressive strength testing under a universal testing machine, and the other 45 were evaluated for sorption and solubility, by first weighing them by a precision weighing scale (W1), then immersing them in artificial saliva for 28 days and weighing them (W2), and finally dehydrating in an oven for 24 hours and weighing them (W3).

**Results:**

Group III (zirconomer) shows the highest compressive strength followed by group II (Miracle Mix) and least compressive strength is seen in group I (glass ionomer cement type IX-Extra) with statistically significant differences between the groups. The sorption and solubility values in artificial saliva were highest for glass ionomer cement type IX - Extra-GC (group I) followed by zirconomer-Shofu (group III), and the least value was seen for Miracle Mix-GC (group II).

**Conclusion:**

Zirconia-reinforced glass ionomer cement is a promising dental material and can be used as a restoration in stress-bearing areas due to its high strength and low solubility and sorption rate. It may be a substitute for silver-reinforced glass ionomer cement due to the added advantage of esthetics.

**Clinical significance:**

This study provides vital information to pediatric dental surgeons on relatively new restorative materials as physical and mechanical properties of the new material are compared with conventional materials to determine the best suited material in terms of durability, strength and dimensional stability.

This study will boost confidence among dental surgeons in terms of handling characteristics, cost effectiveness and success rate.

This study will help clinically and scientifically; pediatric dental surgeons to use this material in stress-bearing areas in pediatric patients.

**How to cite this article:**

Bhatia HP, Singh S, Sood S, Sharma N. A Comparative Evaluation of Sorption, Solubility, and Com-pressive Strength of Three Different Glass Ionomer Cements in Artificial Saliva: An *in vitro* Study. Int J Clin Pediatr Dent 2017;10(1):49-54.

## INTRODUCTION

Dental caries has been considered as a historically important component of the global oral disease burden.

Dental professionals must choose the appropriate material according to the restorative situation. This decision should be based on a number of factors, such as knowledge of the materials’ physical properties, bio-compatibility, esthetics, and application.^1,2^

Glass ionomer cements emerged as a restorative material in the early 1970s by Wilson and Kent et al.^3^ They are esthetically more pleasing than metallic restorations. On the contrary, their use in dentistry as a restorative material in stress-bearing areas is limited due to poor mechanical properties, such as low fracture strength, toughness, and wear resistance.

The solubility of dental restorative materials influences both their rate of degradation and their biological compatibility. The addition of metal powders or fibers to glass ionomer cements can improve its strength. Simmons suggested mixing amalgam alloy powders into the cements and developed this system clinically under the name “Miracle Mix.” This alloy is used for core building and for the treatment of mouths with high caries incidence.^4^ However, their esthetics are poor and they do not take burnish.

A high-strength restorative material, which has been reinforced with zirconia fillers known as zirconomer (white amalgam), has been a recent substitute to glass ionomer cement in dentistry.^5^ Zirconia (ZrO_2_) is a white crystalline oxide of zirconium. It is a polycrystalline ceramic without a glassy phase and exists in several forms. The name “zirconium” comes from the Arabic word “Zargon” which means “golden in color.”.

As it is crucial to have knowledge about the physical and mechanical properties of new products when choosing a restorative material, this *in vitro* study was aimed at comparing the compressive strength, solubility, and sorption of various glass ionomer cements after immersion in artificial saliva.

## MATERIALS AND METHODS

The present study was conducted in the Department of Pedodontics and Preventive Dentistry at Manav Rachna Dental College, Faridabad, Haryana; Research Lab, I Block, Manav Rachna College of Engineering, Faridabad, Haryana; and Spectro Analytical Labs, Okhla Industrial Area, Phase II, New Delhi.

The glass ionomer cements that were used in the study are as follows:

- *Group I:* GC gold label HS posterior extra (type IX) -GC Corporation, Tokyo, Japan
- *Group II:* Miracle Mix (silver alloy glass ionomer cement) - GC Corporation, Tokyo, Japan
- *Group III:* Zirconomer (zirconia-reinforced glass ionomer cement) - Shofu Inc, Kyoto, Japan

**Fig. 1:**
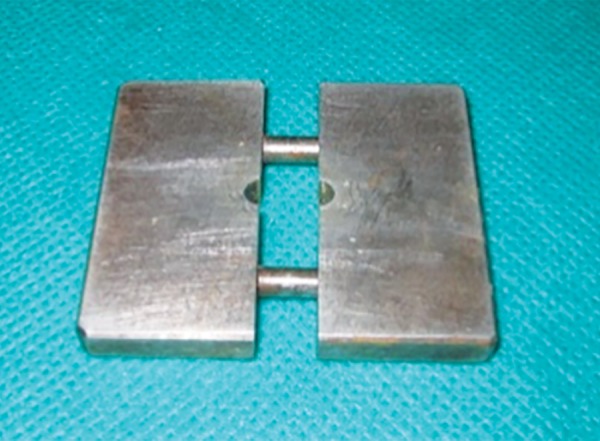
Metal split mold

All the materials were proportioned and manipulated according to the instructions of their respective manufacturers. A metallic split mold with 6 mm of height and 4 mm of diameter was fabricated for the preparation of the specimens (Fig. 1). A total of 90 samples were made.

Petroleum jelly was applied on the inner surfaces of the metal split mold, and it was placed on a mylar strip that was placed on a glass slab. The materials were dispensed onto the mixing pad. The powder and liquid were mixed as per the manufacturer’s instructions, and the mold was slightly overfilled in such a manner to minimize air inclusion. Another mylar strip was placed on the top of the mold and further covered with a second glass slab and pressed for 30 seconds to extrude the excess material and obtain a uniformly smooth specimen surface.

About 1 hour after mixing, the specimens were removed from the mold and any excess material was removed by gentle, dry grinding on both sides with 600-grit silicon carbide paper.

**Fig. 2:**
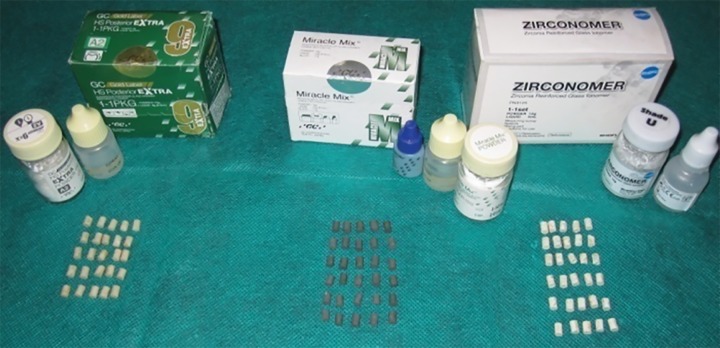
Prepared specimens

## EVALUATION OF SORPTION AND SOLUBILITY IN ARTIFICIAL SALIVA

All specimens were left undisturbed for 30 minutes after preparation (Fig. 2), following which they were subjected to thermocycling test comprising 500 cycles in water between 5°C and 55°C.

The artificial saliva^6^ was prepared in the laboratory from 0.4 gm sodium chloride, 1.21 gm potassium chloride, 0.78 gm sodium dihydrogen phosphate dihydrate (NaH_2_PO_4_ 2H_2_O), 0.005 gm hydrated sodium sulfide (Na_2_S 9H_2_O), 1 gm urea CO (NH_2_)_2_, and 1,000 mL of distilled water. 10 N sodium hydroxide was added to this mixture until the pH value was measured to be as 6.75 ± 0.15.^7^

**Fig. 3:**
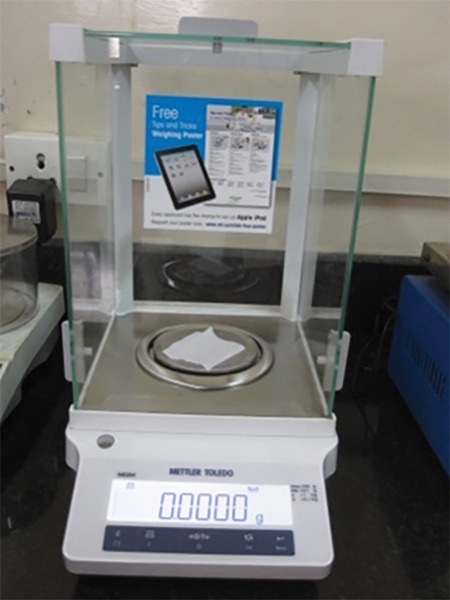
Precision weighing scale

A total of 45 samples (15 of each material) were weighed with precision weighing scale (Fig. 3). The initial weight was termed as W1 (μg).

Immediately after weighing the samples, they were immersed in artificial saliva at pH 7 and stored at 37°C for 28 days.

**Fig. 4:**
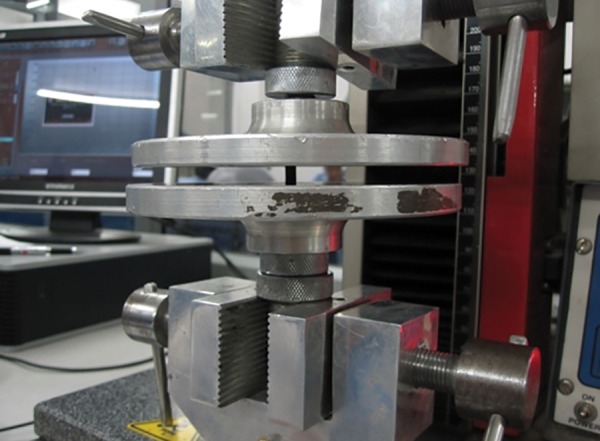
Universal testing machine

After this period, the specimens were removed, washed with water, dried with an absorbent paper, waved in air for 15 seconds, and weighed 1 minute after removal from medium; this weight was termed as W2 (μg).

The samples were then dehydrated in an oven at 37°C for 24 hours and weighed again; this weight was termed as W3 (μg).

Diameter and thickness of each specimen was measured by taking the means of two measurements at right angles to each other made to an accuracy of ±0.01 mm using digital vernier caliper. The volume (V) of each specimen was calculated as follows in cubic millimeters using the mean thickness and diameter:

V = π × r^2^ × h

where r is the mean sample radius (diameter/2) in millimeters and h is the mean sample thickness in millimeters.

The loss of material (solubility) was obtained from the difference between the initial and final drying mass of each sample (W1 - W3). The water sorption was obtained from the difference between initial weighing and the wet weighing (W2 - W1). The values of water sorption (Wsp) and solubility (Wsol), in μg/mm^3^, for each sample were calculated using the following equations:

Wsp = (W2 - W1)/V

Wsol = (W1 - W3)/V

where V is the volume of sample in mm^3^.

## EVALUATION OF COMPRESSIVE STRENGTH

About 45 samples (15 of each material) were immersed in artificial saliva at 37°C at pH 7 for 23 ± 0.5 hours and then subjected to compression strength testing in a universal testing machine (Zwick UTM, Hytech Micro Measurements (P) Ltd, India). The specimens were placed between the plates of the universal testing machine and a compressive load along the long axis was applied at a crosshead speed of 0.5 mm/minute (Fig. 4).

Maximum force at failure was calculated for each specimen and compressive strength was calculated, in MPa, using the following equation^7^:

C = (4p)/(πd^2^)

where p is the maximum force applied, in Newton; d is the average measured diameter of specimen, in mm.

### Statistical Analysis

Analysis of the sorption, solubility, and compressive strength values was performed using one-way analysis of variance (ANOVA) and *post hoc* Dunnett’s T3 test. All statistical analyses were conducted at a significance level of p < 0.05.

## RESULTS

The mean values of Wsorp and the standard deviation for groups I, II, and III were 172.15 ± 17.84, 55.73 ± 6.44, and 75.23 ± 6.76 ug/mm^3^ respectively, and the mean values of Wsol and the standard deviation for groups I, II, and III were 49.71 ± 7.85, 10.70 ± 3.15, and 20.35 ± 5.32 ug/mm^3^ respectively (Table 1).

The sorption and solubility values in artificial saliva were highest for glass ionomer cement type IX - Extra-GC (group I) followed by zirconomer-Shofu (group III) and the least value was seen for Miracle Mix-GC (group II). (p < 0.05) (Table 2).

**Table 1:** **Table 1:** Individual mean values sorption (Wsorp) and solubility (Wsol) with standard deviation for all three groups using one-way ANOVA

										*95% confidence interval for mean*							
				*n*		*Mean*		*Standard deviation*		*Lower bound*		*Upper bound*		*Minimum*		*Maximum*	
Wsorp =		GIC Type IX Extra-GC		15		172.15		17.84		162.27		182.03		124.73		189.75	
W2 - W1/V		Miracle Mix-GC		15		55.73		6.44		52.16		59.30		45.12		69.00	
		Zirconomer-Shofu		15		75.23		6.76		71.49		78.97		63.69		87.57	
		Total		45		101.04		52.72		85.20		116.88		45.12		189.75	
Wsol =		GIC Type IX Extra-GC		15		49.71		7.85		45.36		54.05		30.52		59.71	
W1 - W3/V		Miracle Mix-GC		15		10.70		3.15		8.96		12.45		5.31		15.92	
		Zirconomer-Shofu		15		20.35		5.32		17.40		23.30		11.94		31.85	
		Total		45		26.92		17.70		21.60		32.24		5.31		59.71	

**Graph 1:**
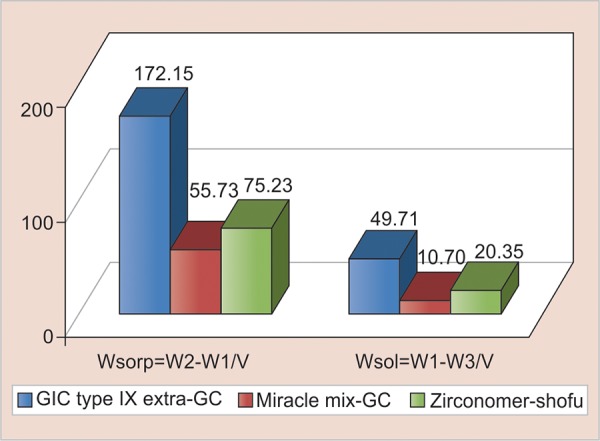
Mean sorption (Wsorp) and solubility (Wsol) values in pg/mm^3^ for groups I, II, and III

These results are presented graphically showing mean sorption and solubility of all the three groups in artificial saliva (Graph 1).

**Graph 2:**
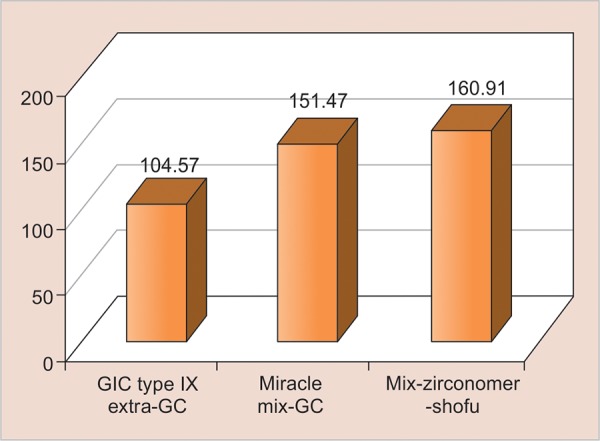
Mean compressive strengths (MPa) for groups I, II, and III

The mean values of compressive strength (MPa) and the standard deviation for groups I, II, and III are 104.57 ± 11.48, 151.47 ± 7.84, and 160.91 ± 8.60 MPa respectively (Table 3). Group III (zirconomer) shows the highest compressive strength followed by group II (Miracle Mix) and least compressive strength is seen in group I (glass ionomer cement type IX-Extra), with statistically significant differences between the groups (Table 4). These results are presented graphically showing mean compressive strengths of all the three groups (Graph 2).

**Table 2:** **Table 2:** Intergroup comparison of sorption (Wsorp) and solubility (Wsol) values after application of *post hoc* Dunnett’s T3 test

*Dependent variable*		*(I) Group*		*(J) Group*		*p-value*	
Wsorp =		GIC Type IX		Miracle Mix-GC		<0.001	
W2 - W1/ V		Extra-GC					
				Zirconomer-Shofu		<0.001	
		Miracle Mix-GC		Zirconomer-Shofu		<0.001	
Wsol =		GIC Type IX		Miracle Mix-GC		<0.001	
W1 - W3/ V		Extra-GC					
				Zirconomer-Shofu		<0.001	
		Miracle Mix-GC		Zirconomer-Shofu		<0.001	

## DISCUSSION

Glass ionomer cements were introduced as hybrids of silicate cements and polycarboxylate cements to have characteristics of fluoride release (from silicate cements) and adhere to enamel and to some extent to dentin (from polycarboxylate cements). It is noteworthy that the physical properties of conventional glass ionomer cement can be highly variable based upon different powder/liquid ratios, so mixing should be adhered to according to the manufacturers’ instructions.^8^

In this study, the zirconia-reinforced glass ionomer cements (white amalgam) have the greatest compres-sive strength (160.91 MPa) owing to the homogeneous incorporation of microsized zirconia particles in the glass component, which further reinforces the material with high strength, lasting durability, and high tolerance to occlusal load. Also, it has the property of transformational toughening, which is the ability to stop the growth of cracks, and it gives zirconia its unique mechanical properties.^9,10^

**Table 3:** **Table 3:** Individual mean values of compressive strength with standard deviation for all three groups using one-way ANOVA

										*95% confidence interval for mean*							
				*N*		*Mean*		*Standard deviation*		*Lower bound*		*Upper bound*		*Minimum*		*Maximum*	
Compressive		GIC Type IX Extra-GC		15		104.57		11.48		98.22		110.93		77.50		119.50	
Strength CS		Miracle Mix-GC		15		151.47		7.84		147.13		155.81		137.70		164.20	
(MPa)		Zirconomer-Shofu		15		160.91		8.60		156.14		165.67		141.00		171.40	
		Total		45		138.98		26.56		131.00		146.96		77.50		171.40	

**Table 4:** **Table 4:** Intergroup comparison of compressive strength values after application of *post hoc* Dunnett T3 test

*(I) Group*		*(J) Group*		*p-value*	
GIC Type IX E		Miracle Mix-GC		<0 .001	
Extra-GC		Zirconomer-Shofu		<0.001	
Miracle Mix-GC		Zirconomer-Shofu		0.012	

Compressive strength of Miracle Mix (151.47 MPa) was much greater than conventional glass ionomer cement as silver particles increased gelation of the cement, but was less than zirconomer due to the fact that simple mixtures of metal powders failed at the metal and poly-acrylate matrix interface and this was the weak link.^11^

The results of this study are in accordance with the studies by Negm et al,^12^ Anstice et al,^13^ Cattani-Lorente et al,^14^ and Dimkov et al.^15^ According to Cattani-Lorente et al,^14^ compressive strength value was more for metal-reinforced glass ionomer cement than conventional Fuji type IX glass ionomer cement.

Kramer and Frankenberger^16^ concluded that metal-reinforced glass ionomer cements are inferior in mechanical properties to conventional glass ionomer cements as clinically tested on posterior class II cavities. Also, according to Yap et al^17^ and Prabhakar et al,^11^ Fuji type IX-Extra had compressive strength comparable to Miracle Mix, and it can act as a substitute for it.^18^

Miracle Mix-GC has the lowest sorption and solubility values in artificial saliva, i.e., 55.73 and 10.7 ug/mm^3^ respectively, with respect to Fuji type IX-Extra and zir-conomer. The addition of silver had the advantage of increasing radiopacity of the cements and forming strong bonds with the matrix, which is responsible for lower sorption and solubility of the material.

A study by Meşe et al^19^ stated that sorption and solubility values were found to depend on the type and content of filler, filler concentration, mean particle size, the coupling agents, the nature of the filler particles, and the type of solvent.

Benhameurlain et al^20^ stated that conventional glass ionomer cement showed greatest sorption and material net loss as compared to other tooth-colored restorative materials.According to Sidhu et al,^21^ the modified glass ionomer cement has similar or comparable water balance as conventional glass ionomer cements and, hence, is equally moisture sensitive.

Since glass ionomer cements are widely accepted, further characterization of the effects of water on glass ionomer cements is warranted. More studies should be directed toward the evaluation of effects of saliva on the long-term clinical durability of conventional and modified glass ionomer cements.

## CONCLUSION

This study elucidated essential values for the evaluation of the quality of each employed material, which is of important clinical applicability.

The materials investigated in this study demonstrated different levels of sorption, solubility, and compressive strength. According to the results, following conclusions can be drawn:

- Zirconia-reinforced glass ionomer cement has the highest compressive strength among all the groups tested; silver-reinforced glass ionomer cement has the second highest value, and the least value of com-pressive strength is seen with glass ionomer cement type IX-Extra, and the differences are statistically significant.
- Silver-reinforced glass ionomer cement absorbed less water and is less soluble than other cements followed by zirconia-reinforced glass ionomer cement, and the highest sorption and solubility rates in artificial saliva were seen with glass ionomer cement type IX-Extra. There were significant differences among the tested materials.
- Zirconia-reinforced glass ionomer cement is a promising dental material and can be used as a restoration in stress-bearing areas due to its high strength and low solubility and sorption rates. It may be a substitute for silver-reinforced glass ionomer cement due to the added advantage of esthetics.
